# Solid-variant primary pulmonary adenoid cystic carcinoma with pleural metastasis and malignant pleural effusion: a rare case report

**DOI:** 10.3389/fonc.2026.1827108

**Published:** 2026-05-18

**Authors:** Suyi Guo, Yang Zhai, Hongbian Gao, Caixia Ding

**Affiliations:** 1 Shaanxi University of Chinese Medicine, Xianyang, China; 2 Department of Medical Oncology, Shaanxi Provincial Cancer Hospital, Xi’an, China; 3 Department of Pathology, Shaanxi Provincial Cancer Hospital, Xi’an, China

**Keywords:** CAP-like chemoimmunotherapy, diagnostic challenge, immune checkpoint inhibitor, pleural metastasis, primary pulmonary adenoid cystic carcinoma, solid-variant, SOX10

## Abstract

**Background:**

Pulmonary adenoid cystic carcinoma (PACC) is a rare salivary gland-type malignancy, accounting for 0.04%–0.2% of primary lung neoplasms. The solid variant with pleural metastasis and malignant pleural effusion is exceptionally uncommon, posing significant diagnostic and therapeutic challenges.

**Case presentation:**

We report a 65-year-old man whose lung biopsy was initially interpreted as squamous cell carcinoma without keratinization in the setting of basaloid morphology and p40/p63 positivity. The diagnosis was revised to solid-variant primary PACC after an expanded immunohistochemical workup demonstrated biphasic epithelial–myoepithelial differentiation, including expression of SOX10 and c-Myb. Given the advanced stage, the patient received a systemic combination of tislelizumab, cyclophosphamide, pegylated liposomal doxorubicin, and nedaplatin, initially combined with local intrapleural therapy. This treatment was followed by a partial response and marked symptomatic improvement without grade ≥3 treatment-related adverse events.

**Conclusion:**

This case highlights a diagnostic pitfall in lung tumors exhibiting squamoid immunophenotypes and underscores the necessity of incorporating myoepithelial markers into the diagnostic workup. Furthermore, it provides a cautiously interpreted clinical observation of immune checkpoint inhibitor–based combination therapy in advanced PACC.

## 1 Introduction

Primary pulmonary adenoid cystic carcinoma (PACC) is a rare salivary gland–type malignancy of the lung that is recognized as one of the primary salivary gland–type tumors in the 2021 World Health Organization (WHO) classification of lung tumors and predominantly involves the central airways, including the trachea and main bronchi (1–5). PACC is thought to arise from the minor salivary glands distributed along the tracheobronchial tree and is typically characterized by indolent growth and low-grade malignant behavior (1, 3, 4). Metastatic spread most commonly involves the regional lymph nodes, lungs, bones, liver, skin, and brain (1, 3, 4). In contrast, pleural metastasis accompanied by malignant pleural effusion has only rarely been reported in the adenoid cystic carcinoma literature (6–9). Histologically, PACC exhibits three principal growth patterns: tubular, cribriform, and solid (2, 4). Among these, the solid variant is distinguished by densely cellular, sheet-like growth with a marked loss of typical glandular architecture and has been associated with more aggressive behavior and unfavorable clinical outcomes (4). Owing to its biphasic composition of ductal epithelial and myoepithelial/basal cell components, PACC can show p63 and p40 expression, thereby creating immunophenotypic overlap with basaloid squamous cell carcinoma and making the distinction between solid-variant PACC and squamous cell carcinoma without keratinization challenging, particularly in small biopsy specimens (4, 10). We report the case of a 65-year-old man whose lung biopsy was initially interpreted as squamous cell carcinoma without keratinization. After expert pathology consultation, the diagnosis was revised to solid-variant primary PACC based on the integrated morphologic and immunophenotypic findings, including evidence of biphasic epithelial–myoepithelial differentiation and expression of SOX10, CD117, and c-Myb. The patient presented with pleural metastasis and malignant pleural effusion and was classified as having stage IV disease. At present, evidence-based therapeutic strategies for advanced PACC remain extremely limited, and no standard systemic treatment has been established. By describing this exceptionally rare case of solid-variant PACC with pleural metastasis, we aim to highlight key diagnostic pitfalls in immunohistochemical differentiation and to provide preliminary clinical evidence on the feasibility of chemoimmunotherapy (tislelizumab combined with chemotherapy) as a systemic palliative approach for advanced disease.

## 2 Case presentation

**Figure 1:**
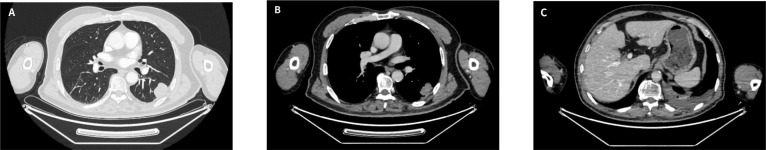
Baseline chest CT findings. **(A)** Lung window showing a mass-like lesion measuring approximately 3.2cm in diameter in the dorsal segment of the left lower lobe, accompanied by linear opacities in the right lower lobe. **(B)** Mediastinal window showing the left lung mass with adjacent pleural thickening and a small volume of pleural effusion. **(C)** Mediastinal window at the abdominal level showing a small amount of left-sided pleural effusion superior to the spleen.

A 65-year-old man was admitted to our hospital in October 2024 for the evaluation of a rapidly progressing left lower-lobe lung mass. The patient’s clinical course began in April 2024, when a routine health examination at an outside hospital detected a solitary nodule measuring approximately 0.97cm in the left lower lobe. A follow-up chest computed tomography (CT) scan in September 2024 demonstrated a marked increase in the lesion’s size to 2.8cm. On admission in October, physical examination revealed dullness to percussion and decreased breath sounds over the left lower lung field. The patient reported mild dyspnea on exertion but denied cough, hemoptysis, fever, or noticeable weight loss. Tumor marker profiling revealed a ferritin level of 19.31 ng/mL and a cytokeratin 19 fragment (CYFRA 21-1) level of 3.86 ng/mL (indicating a slight elevation); other routine laboratory results were unremarkable. His past medical history was notable for hypopharyngeal carcinoma of the right piriform recess diagnosed in March 2018. According to the available historical pathology consultation report, the tumor had been diagnosed as moderately to poorly differentiated squamous cell carcinoma based on morphology and the original immunohistochemical findings. He received five cycles of chemoradiotherapy and remained under regular follow-up without evidence of recurrence. Although no clinical recurrence had been documented, this prior history was considered in the initial differential diagnosis of the current lung lesion. The subsequent clinicoradiologic and pathologic findings, including PET/CT assessment and expert pathology consultation, were more consistent with a primary pulmonary salivary gland–type tumor than with recurrent or metastatic hypopharyngeal squamous cell carcinoma. He also had a 6-year history of hypertension (historically up to 180/110 mmHg, currently well-controlled on olmesartan) and a 2-year history of type 2 diabetes mellitus (well-controlled on acarbose and metformin). Additionally, he had a history of cerebral infarction in 2022, which was managed with bilateral carotid artery stenting and currently maintained on clopidogrel and atorvastatin. Regarding his social and family history, he was a lifelong never-smoker, but his family history was significant for a brother who died of lung cancer. A repeat chest CT performed at our hospital on October 21, 2024, revealed further progression: multiple subpleural soft-tissue nodules were now present in the left hemithorax, with the largest measuring approximately 3.2cm in diameter, consistent with the left lower-lobe mass. Additionally, linear opacities were observed in the right lower lobe. Left pleural thickening and a moderate pleural effusion had newly developed (**Figure 1**), findings suspicious for an aggressive lung malignancy with malignant pleural involvement, pending cytopathological confirmation.

**Figure 2:**
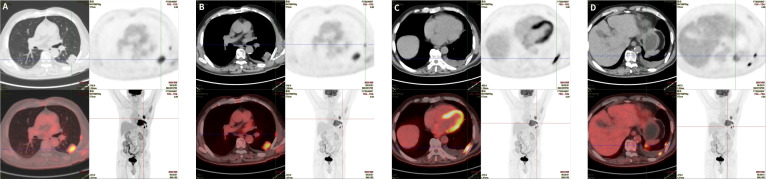
PET/CT findings. **(A)** Hypermetabolic mass-like lesion in the dorsal segment of the left lower lobe with markedly increased FDG uptake (SUV_max_, 9.07), suggestive of a peripheral lung malignancy with invasion of the adjacent pleura. **(B, C)** Multiple focal thickenings of the left parietal and visceral pleura showing increased FDG uptake (SUV_max_ range, 3.40–13.28), consistent with pleural metastases. **(D)** Focal increased FDG uptake in the anterior portion of the left seventh rib, suspicious for bone metastasis.

An ^18^F-fluorodeoxyglucose (^18^F-FDG) positron emission tomography/computed tomography (PET/CT) scan performed on October 23, 2024 demonstrated a hypermetabolic mass in the dorsal segment of the left lower lobe, suggestive of a peripheral lung malignancy with invasion of the adjacent pleura. Multiple areas of focal thickening involving the left parietal and visceral pleura, as well as a linear opacity in the left pleural recess, showed increased FDG uptake, concerning for pleural metastases (**Figure 2**). A moderate left pleural effusion was also present. No additional primary tumor was identified on PET/CT, and there was no evidence of tumor in the major salivary glands or salivary gland region of the head and neck.

To obtain histological confirmation, the patient underwent a CT-guided percutaneous lung biopsy on October 28, 2024. The initial histopathological examination of the biopsy specimen revealed basaloid morphology suggestive of squamous cell carcinoma without keratinization. Immunohistochemical (IHC) staining demonstrated positive expression of p40, p63, and CK5/6, alongside focal positivity for CD56 and a high Ki-67 proliferation index of approximately 70%. Thyroid transcription factor-1 (TTF-1) was negative, as were Napsin A, CK7, Syn, and CgA. Based on these initial findings, a diagnosis of squamous cell carcinoma without keratinization was initially favored. No definite perineural invasion or unequivocal lymphovascular invasion was identified in the limited biopsy specimen. On retrospective review of the initial H&E-stained sections, focal tubular/cribriform-like structures and basement-membrane-like/hyaline material were noted. The biopsy was predominantly composed of basaloid solid nests, a pattern that was later considered compatible with solid-variant PACC when interpreted together with the extended immunohistochemical profile and expert pathology consultation.

**Figure 3:**
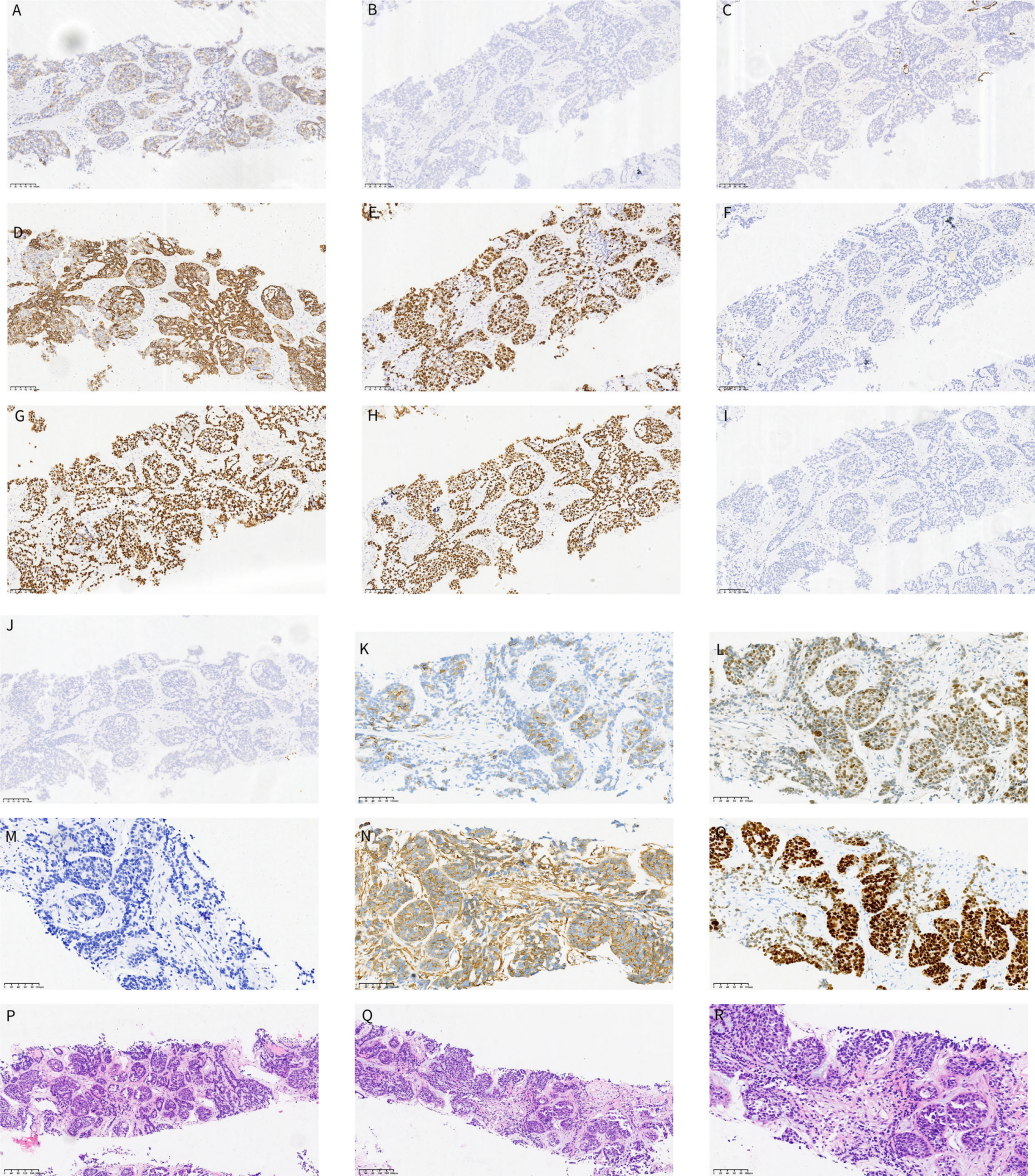
Representative histopathological and immunohistochemical findings of the lung biopsy specimen (Part 1). Immunohistochemical staining showed: **(A)** CD56, focal positivity; **(B)** CgA, negative; **(C)** CK7, negative; **(D)** CK5/6, diffuse positive expression; **(E)** Ki-67, high proliferation index (approximately 70%); **(F)** Napsin A, negative; **(G)** p40, strong nuclear positivity; **(H)** p63, strong nuclear positivity; **(I)** Syn, negative. The strong positivity for p40, p63, and CK5/6 initially suggested a diagnosis of squamous cell carcinoma without keratinization. Representative histopathological and immunohistochemical findings of the lung biopsy specimen (Part 2). Immunohistochemical staining showed: **(J)** TTF-1, negative; **(K)** CD117, partial positivity, predominantly in luminal/ductal-type tumor cells; **(L)** c-Myb, nuclear positivity in tumor cells; **(M)** S100, focal positivity; **(N)** SMA, partial positivity in abluminal myoepithelial/basal-type cells; **(O)** SOX10, positive staining predominantly in abluminal myoepithelial/basal-type cells; **(P, Q)** hematoxylin and eosin (H&E) staining, low-power views (×10), showing solid nests of basaloid tumor cells with focal basement-membrane-like/hyaline material; **(R)** H&E staining, higher-power view (×20). Partial CD117 positivity supported a luminal/ductal component, whereas SOX10, S100, and SMA supported abluminal myoepithelial/basal differentiation. Together with nuclear c-Myb positivity and CK8/18 positivity documented in the outside consultation report, these findings supported biphasic epithelial–myoepithelial differentiation and, in conjunction with the morphologic findings, the diagnosis of solid-variant primary pulmonary adenoid cystic carcinoma. The CK8/18-stained slide or image was not available among the consultation materials accessible to the authors and therefore could not be included in this figure panel.

Given the atypical peripheral presentation with suspected pleural involvement and the morphologic differential diagnosis of solid-variant adenoid cystic carcinoma, an extended IHC workup was performed, and the case was subsequently submitted for expert pathology consultation to clarify the diagnosis and avoid a potential diagnostic pitfall. The outside consultation report documented positive expression of CK8/18, SOX10, and c-Myb, with partial positivity for CD117 (clone YR145), smooth muscle actin (SMA), and calponin, as well as focal positivity for S100. GFAP was negative. CK7 had been included in the initial immunohistochemical panel and was negative; therefore, CK7 was not used as evidence of luminal differentiation. On the extended immunohistochemical panel, luminal/ductal differentiation was supported by CK8/18 positivity documented in the consultation report and by partial CD117 expression in the available stained material, whereas SOX10 positivity, focal S100 positivity, and partial SMA and calponin expression highlighted abluminal myoepithelial/basal-type cells. The CK8/18-stained image or slide was not provided with the outside consultation material and therefore could not be included in the figure panel; accordingly, CK8/18 is described in the text but not illustrated in **Figure 3**. c-Myb showed nuclear positivity in tumor cells, further supporting adenoid cystic carcinoma. Notably, p53 showed diffuse strong overexpression, consistent with a mutant-pattern staining result, and PD-L1 expression, evaluated using the 22C3 companion diagnostic assay, yielded a combined positive score (CPS) of <1. In conjunction with the expert consultation, the integrated morphologic and immunophenotypic findings supported biphasic epithelial–myoepithelial differentiation and led to the revised diagnosis of solid-variant primary pulmonary adenoid cystic carcinoma (PACC). Representative histopathological and immunohistochemical findings are shown in **Figure 3**.

To explore potential targeted therapeutic opportunities, next-generation sequencing (NGS)–based circulating tumor DNA (ctDNA) profiling was performed using 10 mL of peripheral blood collected in a Streck tube on November 13, 2024. The assay was performed by DIAN Diagnostics (Hangzhou, China) using a targeted 16-gene lung cancer panel. The NGS results (reported on November 22, 2024; **Table 1**) revealed a TP53 missense mutation (p.C176F) with a low ctDNA variant allele frequency (VAF) of 2.87%; no actionable driver mutation was identified within the scope of this panel. Because the assay was blood-based rather than tissue-based, this VAF should not be interpreted as tumor tissue allele frequency. The report provided overall sequencing quality metrics, including a mean DNA sequencing depth of 4727.74× and DNA 1000× coverage of 94.14%, but did not provide TP53 locus-specific coverage depth or the number of variant-supporting reads. Therefore, the TP53 finding was interpreted cautiously and was not used as independent evidence for tumor classification.

**Table 1:** Summary of blood-based ctDNA profiling results from the targeted lung cancer panel.

Gene	Variant type	Result
TP53	SNV	p.C176F, VAF 2.87%; non-actionable
ALK	SNV,Fusion	Not Detected
BRAF	SNV,Indel	Not Detected
EGFR	SNV,Indel	p.R324H, VAF 0.27%; VUS, non-actionable
ERBB2	SNV,Indel,CNV	Not Detected
KRAS	SNV,Indel	Not Detected
MET	SNV,Indel,CNV	Not Detected
NTRK1	Fusion	Not Detected
NTRK2	Fusion	Not Detected
NTRK3	Fusion	Not Detected
RET	Fusion	Not Detected
ROS1	Fusion	Not Detected

The assay was performed on peripheral blood ctDNA. TP53 locus-specific coverage depth and variant-supporting read counts were not provided in the available molecular report. VAF, variant allele frequency; VUS, variant of uncertain significance; SNV, single-nucleotide variant; Indel, insertion/deletion; CNV, copy number variation.

Integrating the clinical presentation, PET/CT findings, outside expert pathology consultation, and histopathological and immunohistochemical evidence, the patient was diagnosed with stage IVA (cT2aNxM1a) solid-variant PACC with pleural metastasis and malignant pleural effusion. PET/CT did not identify another primary site and showed no evidence of tumor in the major salivary glands or salivary gland region of the head and neck. Lymph node status was assessed radiologically on chest CT and PET/CT only; no invasive nodal sampling was performed. Accordingly, the nodal category was recorded as cNx.

During the diagnostic workup period in November 2024, as the disease progressed and the left pleural effusion worsened, the patient gradually developed clinical symptoms, including new-onset cough, sputum production, and occasional blood-streaked sputum. To manage the pleural effusion and obtain further diagnostic evidence, ultrasound-guided placement of a left pleural drainage catheter was performed. Cytological examination of the pleural fluid revealed clusters of malignant cells. The cytomorphological features and immunohistochemical profile were completely concordant with those of the primary lung lesion, definitively confirming a malignant pleural effusion attributable to pleural metastasis. Consequently, the disease was formally staged as stage IVA, rendering the patient unsuitable for surgical resection.

Given the advanced stage, the rarity of the disease, and the absence of an established standard first-line systemic therapy for advanced primary pulmonary adenoid cystic carcinoma (PACC), the case was discussed at a multidisciplinary team (MDT) meeting. An empirically selected, individualized palliative treatment strategy was formulated by the institutional MDT. This approach should be considered exploratory, as no validated regimen exists for advanced PACC. Rather than following a standard thoracic oncology regimen, the systemic treatment strategy was informed by limited historical experience with cyclophosphamide-, doxorubicin-, and platinum-containing regimens in adenoid cystic carcinoma (ACC), including CAP-like approaches, as reported in small studies and contemporary reviews or practical guidance (11–14). However, the available evidence remains limited, is derived predominantly from salivary gland ACC, and does not establish a standard treatment approach for PACC (12–14). Accordingly, the present regimen should be regarded as an extrapolated, MDT-selected strategy rather than a standard treatment approach supported by disease-specific evidence. In this context, cyclophosphamide and pegylated liposomal doxorubicin were incorporated into an exploratory CAP-like chemoimmunotherapy regimen. Tislelizumab was added empirically despite a PD-L1 combined positive score (CPS) of <1, reflecting the absence of an established immunotherapy-based strategy for advanced PACC. Nedaplatin was selected as the platinum component of this adapted regimen primarily in consideration of anticipated tolerability in an older patient with multiple comorbidities, rather than because of any established role in PACC.

First-line treatment was initiated on November 30, 2024, and the 6-cycle induction phase was completed on May 7, 2025. During cycle 1, the patient received systemic therapy consisting of tislelizumab (200 mg), pegylated liposomal doxorubicin (50 mg), and cyclophosphamide (1g). Concurrently, following adequate pleural drainage, intrapleural perfusion therapy was administered using nedaplatin, recombinant human endostatin, interleukin-2 (IL-2), and dexamethasone. A marked reduction in pleural effusion was observed after this initial cycle, as evidenced by decreased drainage volume and repeat ultrasound assessment. Based on this favorable local response, intrapleural therapy was discontinued after cycle 1. From cycles 2 to 6, nedaplatin (60 mg on days 1–2 per cycle) was incorporated into the systemic regimen alongside tislelizumab, cyclophosphamide, and pegylated liposomal doxorubicin. Following completion of induction therapy, the patient transitioned to maintenance tislelizumab monotherapy at 200 mg every 21 days. Additionally, denosumab was administered as bone-modifying therapy in the context of the suspected rib metastasis identified on PET/CT.

**Figure 4:**
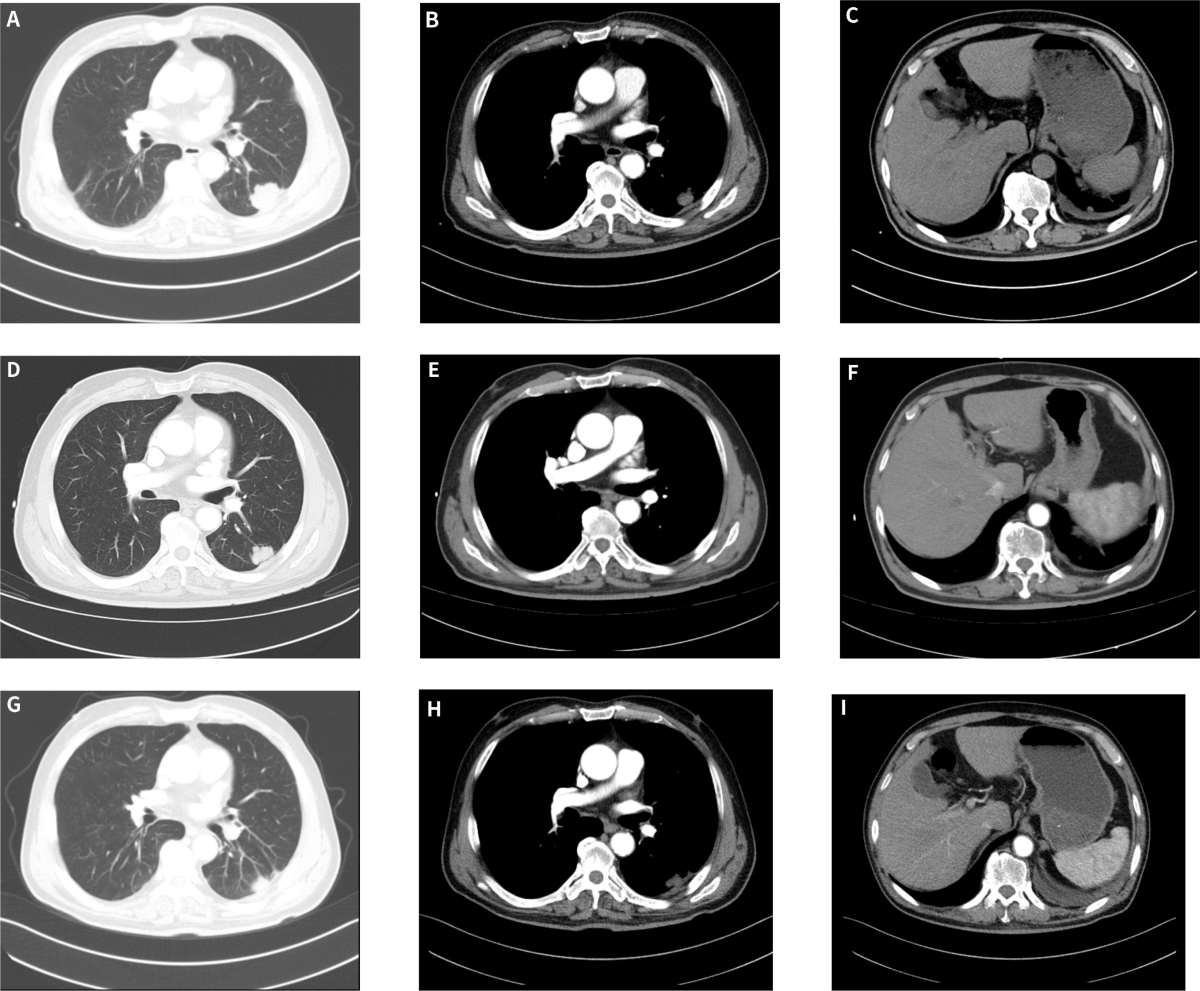
Representative chest computed tomography (CT) images following multiple cycles of treatment. **(A)** Lung window after 2 cycles of treatment; **(B, C)** Mediastinal window after 2 cycles of treatment. **(D)** Lung window after 4 cycles of treatment; **(E, F)** Mediastinal window after 4 cycles of treatment. **(G)** Lung window after 6 cycles of treatment; **(H, I)** Mediastinal window after 6 cycles of treatment.

**Figure 5:**
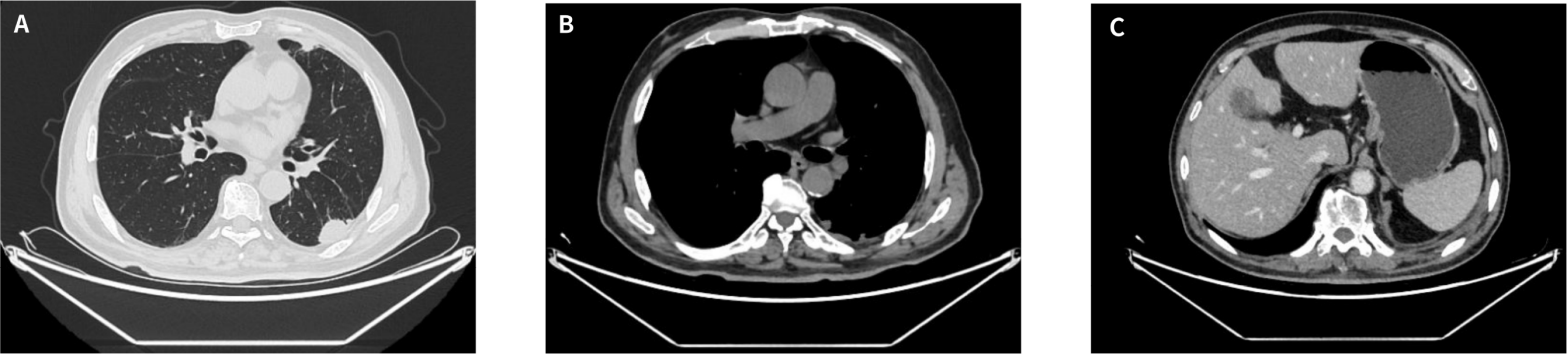
Follow-up chest CT obtained on December 31, 2025, during maintenance tislelizumab monotherapy. **(A)** Lung window image showing persistent reduction of the left lower-lobe pulmonary lesion compared with baseline. **(B)** Mediastinal window image showing no radiologic progression of residual pleural/subpleural lesions. **(C)** Mediastinal window image at the upper abdominal level showing no recurrent or increased left pleural effusion. Overall, compared with baseline, the findings are consistent with an ongoing partial response without evidence of local progression.

Despite multiple baseline comorbidities, including hypertension, type 2 diabetes mellitus, and sequelae of cerebral infarction, the patient tolerated the regimen well, and no treatment-related adverse events of grade ≥3 were observed. Serial radiologic measurements are summarized in **Table 2**. According to RECIST version 1.1 (15), the patient had stable disease after 2 cycles, achieved a partial response after 4 cycles, and maintained partial response after 6 cycles (**Figure 4**). At maintenance follow-up on December 31, 2025, the principal measurable lesion remained reduced compared with baseline and did not meet criteria for progression, consistent with ongoing partial response. The pleural and subpleural non-target lesions showed no unequivocal progression, and no new distant metastases were identified. This radiologic improvement was accompanied by marked improvement in clinical symptoms, including cough, sputum production, and occasional blood-streaked sputum. A follow-up chest CT performed on December 31, 2025 (**Figure 5**) confirmed ongoing partial response during maintenance therapy. The patient remains on outpatient follow-up and continues to receive maintenance tislelizumab monotherapy at 200 mg every 21 days. As of the last clinical follow-up on March 5, 2026, the patient was alive, remained on this maintenance regimen, and had no clinical evidence of disease progression.

**Table 2:** Serial radiologic assessment of treatment response according to RECIST version 1.1.

Time point	Date	Longest diameter (cm)	Sum of diameters (cm)	% change from baseline	RECIST assessment
Baseline	2024-10-21	3.2	3.2	Reference	Baseline
After 2 cycles	2025-01-27	2.9	2.9	-9.4%	Stable disease
After 4 cycles	2025-04-02	2.1	2.1	-34.4%	Partial response
After 6 cycles	2025-05-07	1.3	1.3	-59.4%	Partial response
Maintenance follow-up	2025-12-31	1.5	1.5	-53.1%	Partial response

Response was assessed on serial chest CT according to RECIST version 1.1. The principal measurable lesion in the left lower lobe was used as the target lesion for quantitative assessment, whereas pleural disease and subpleural nodules were followed qualitatively as non-target disease. Because only one target lesion was used, the sum of diameters was identical to the longest diameter of that lesion.

## 3 Discussion

Primary pulmonary adenoid cystic carcinoma (PACC) is a rare salivary gland–type tumor of the lung. Histologically, PACC is classically categorized into three growth patterns—cribriform, tubular, and solid—of which the solid pattern is the least common but is associated with more aggressive biological behavior and a higher likelihood of lymph node and distant metastases (16).

In this report, we describe a case of solid-variant PACC presenting with pleural metastasis and malignant pleural effusion, an exceptionally rare clinical manifestation. This unusual presentation highlights the substantial heterogeneity of solid-variant PACC in terms of radiological appearance and disease progression, and underscores the diagnostic and therapeutic challenges associated with this aggressive subtype.

### 3.1 Morphologic and immunohistochemical challenges

Solid-variant PACC can closely resemble basaloid squamous cell carcinoma or neuroendocrine carcinoma in limited biopsy specimens, particularly when the initial immunohistochemical panel does not clearly demonstrate dual luminal and abluminal differentiation. In the present case, a diagnosis of squamous cell carcinoma without keratinization was initially favored because the biopsy showed basaloid morphology with p40, p63, and CK5/6 positivity. The patient’s prior history of squamous cell carcinoma of the right pyriform recess also reasonably entered the initial clinical differential diagnosis. On retrospective review after expert consultation, focal basement-membrane-like/hyaline material within the tumor nests was recognized as a subtle morphologic feature compatible with adenoid cystic carcinoma, but this finding alone was not sufficient to establish the diagnosis in the limited biopsy specimen. The diagnosis was ultimately revised after the extended immunohistochemical workup demonstrated epithelial–myoepithelial differentiation, including SOX10 positivity in abluminal myoepithelial/basal-type cells and nuclear c-Myb positivity in tumor cells.

SOX10 is a key transcription factor involved in the differentiation of neural crest–derived cells and salivary gland stem/progenitor cells (17). Its expression provides strong evidence of myoepithelial differentiation and supports classification of the tumor within the salivary gland–type spectrum (18). More broadly, SOX family transcription factors have been implicated in lineage specification and tumor differentiation across multiple cancer types, further underscoring their fundamental role in tumor biology (19).

In addition, previous studies have demonstrated that strong and diffuse (3+) nuclear staining for c-Myb is a characteristic feature of solid or basaloid adenoid cystic carcinoma, whereas this staining pattern is rarely encountered in other poorly differentiated malignancies (20, 21).

Notably, CD117 (c-KIT) may serve as a useful adjunctive marker in the diagnosis of PACC, particularly when interpreted together with c-Myb expression and the overall morphologic and immunophenotypic findings (22–24).

Squamous cell carcinoma without keratinization may closely mimic solid-variant PACC on morphologic examination and frequently exhibits immunoreactivity for p63 and CK5/6. However, CD117 expression is typically absent in squamous cell carcinoma without keratinization, rendering CD117 a useful marker for distinguishing between these two entities (24).

In the present case, SOX10 and nuclear c-Myb expression, together with partial CD117 positivity and the extended biphasic immunophenotype described above, supported the diagnosis of solid-variant PACC. The blood-based 16-gene ctDNA panel did not identify actionable NSCLC driver alterations; however, this result was considered relevant to therapeutic profiling rather than diagnostic confirmation, given the limited gene coverage and blood-based nature of the assay. The final diagnosis was therefore based on the integrated morphologic, immunophenotypic, clinicoradiologic, and expert consultation findings.

### 3.2 MYB family and NOTCH signaling pathway

Recurrent MYB gene rearrangements represent a hallmark molecular alteration in primary pulmonary adenoid cystic carcinoma (PACC) and constitute a key feature distinguishing this entity from other salivary gland–type lung tumors (25). Notably, MYB and MYBL1 encode proteins with nearly identical DNA-binding domains and highly similar overall structures, supporting the concept that these MYB family members function as interchangeable oncogenic drivers in adenoid cystic carcinoma (26, 27).

In addition to MYB alterations, recent genomic studies have identified mutations involving the NOTCH signaling pathway in a subset of PACC cases, most frequently affecting NOTCH1 (28), and less commonly NOTCH2–4, as well as downstream signaling components such as LPAR3 and ALPI (25, 29). These findings underscore the molecular heterogeneity of PACC and highlight the importance of comprehensive molecular profiling to avoid misclassification (26).

The molecular findings in this case were interpreted cautiously. The targeted ctDNA panel did not identify actionable NSCLC driver alterations within the scope of the assay, and the low-VAF TP53 p.C176F variant was not used for tumor classification or prognostic interpretation. The aggressive clinicopathologic profile was instead assessed on the basis of the observed features, including pleural metastasis, advanced stage, and a high Ki-67 labeling index of approximately 70%. A high Ki-67 labeling index has been associated with increased proliferative activity and adverse clinical behavior in adenoid cystic carcinoma (26).

However, a major methodological limitation of the present case should be acknowledged. Although recurrent MYB/MYBL1 rearrangements are widely regarded as the molecular gold standard for confirming adenoid cystic carcinoma, including PACC (25–27), no tissue-based fusion assay, such as fluorescence *in situ* hybridization (FISH), targeted RNA sequencing, or DNA-based fusion testing, was performed in this case. The 16-gene blood-based ctDNA panel was obtained for therapeutic genomic profiling and did not interrogate MYB, MYBL1, or NFIB. In addition, the low-VAF TP53 p.C176F finding was interpreted cautiously because TP53 locus-specific coverage depth and variant-supporting read counts were not provided in the available molecular report. No suitable residual tumor tissue was available for further tissue-based fusion testing after routine histologic examination and extended immunohistochemical workup. Accordingly, the diagnosis in this patient rests on an integrated assessment of morphologic, immunohistochemical, and clinicoradiologic findings rather than on molecular confirmation. Overall, these findings were considered strongly supportive of a diagnosis of solid-variant PACC.

### 3.3 Exploratory use of CAP-like chemoimmunotherapy in advanced PACC

Systemic treatment options for adenoid cystic carcinoma (ACC), particularly primary pulmonary adenoid cystic carcinoma (PACC), remain limited, and no established standard first-line systemic therapy is available for advanced disease. Complete surgical resection remains the cornerstone of treatment and offers the best chance for durable disease control in patients with localized tumors (1–3, 30). However, therapeutic options are particularly constrained in patients with pleural dissemination or in those who are not candidates for surgery. Importantly, most currently available evidence regarding systemic treatment is extrapolated from recurrent or metastatic salivary gland ACC rather than from pulmonary primary tumors (12, 13, 26). Recent real-world data further highlight the heterogeneity of treatment patterns and the absence of consensus regarding optimal systemic therapy in metastatic ACC (14).

Historically, cytotoxic chemotherapy has shown only modest activity in ACC. Among the reported regimens, combinations containing cyclophosphamide, anthracyclines, and platinum agents—including the CAP regimen (cyclophosphamide, doxorubicin, and cisplatin)—have been described, although the supporting evidence remains limited and is derived largely from small retrospective series (11–13). In the present case, the inclusion of cyclophosphamide and pegylated liposomal doxorubicin was informed by this prior ACC experience. Nedaplatin was selected instead of cisplatin as an empirical modification of a CAP-like regimen, primarily in consideration of anticipated tolerability in an older patient with multiple comorbidities, rather than on the basis of disease-specific evidence in PACC. Accordingly, the overall treatment strategy should be interpreted as an exploratory CAP-like chemoimmunotherapy regimen selected by the institutional multidisciplinary team (MDT), rather than as a validated standard approach for advanced PACC.

From an immunobiological perspective, ACC is generally regarded as an immunologically “cold” tumor, with sparse tumor-infiltrating lymphocytes and low or absent PD-L1 expression reported in prior studies (26, 31). In line with these features, the clinical efficacy of immune checkpoint inhibitors in ACC has not been systematically established (32). The incorporation of PD-1 blockade in the present case therefore also reflects an exploratory strategy. Although the patient achieved a sustained partial response despite a PD-L1 combined positive score (CPS) of <1, the biological basis of this response remains uncertain. One possible explanation is that systemic chemotherapy and/or local intrapleural therapy may have altered the tumor microenvironment in a way that enhanced antigen release or immune recognition. However, in the absence of post-treatment tumor tissue, tumor mutational burden assessment, or dedicated immune profiling, any hypothesis involving immunogenic cell death or immune reprogramming must remain speculative.

Taken together, this case suggests that an individualized, MDT-guided CAP-like chemoimmunotherapy strategy may be feasible in selected patients with advanced PACC, but the findings require cautious interpretation. Given the lack of an established standard systemic therapy and the exploratory nature of the present regimen, this experience should be regarded as hypothesis-generating rather than as evidence supporting a validated treatment paradigm.

## 4 Conclusion

We report a rare case of solid-variant primary pulmonary adenoid cystic carcinoma (PACC) with pleural metastasis and malignant pleural effusion. This case highlights the substantial diagnostic challenges posed by solid-variant PACC, which may closely mimic squamous cell carcinoma without keratinization owing to overlapping morphologic features and immunophenotypic expression, including frequent p40 and p63 positivity. In such atypical presentations, an expanded immunohistochemical panel covering luminal/ductal differentiation, myoepithelial/basal differentiation, and c-Myb expression is essential to achieve accurate tumor classification.

From a therapeutic perspective, this patient with advanced pleural dissemination who was not eligible for curative surgery achieved a partial response following treatment with tislelizumab in combination with chemotherapy. This observation suggests that chemoimmunotherapy may represent a potential treatment option in selected patients with advanced PACC; however, given that the evidence is derived from a single case, it should not be generalized or regarded as a standard therapeutic approach.

Looking ahead, we advocate for comprehensive molecular and pathological evaluation in similar cases, with particular emphasis on MYB/MYBL1 fusion status, alterations in the NOTCH signaling pathway, and potential predictive biomarkers such as PD-L1 expression and tumor mutational burden (TMB). Such integrated profiling may facilitate the identification of rational therapeutic targets and clinical trial opportunities, and help to validate the findings observed in this case in larger cohorts or prospective studies.

## Data Availability

The original contributions presented in the study are included in the article/Supplementary Material. Further inquiries can be directed to the corresponding author.
