# Correction: Solid-variant primary pulmonary adenoid cystic carcinoma with pleural metastasis and malignant pleural effusion: a rare case report

**DOI:** 10.3389/fonc.2026.1929751

**Published:** 2026-07-21

**Authors:** Suyi Guo, Yang Zhai, Hongbian Gao, Caixia Ding

**Affiliations:** 1 Shaanxi University of Chinese Medicine, Xianyang, China; 2 Department of Medical Oncology, Shaanxi Provincial Cancer Hospital, Xi’an, China; 3 Department of Pathology, Shaanxi Provincial Cancer Hospital, Xi’an, China

**Keywords:** CAP-like chemoimmunotherapy, diagnostic challenge, immune checkpoint inhibitor, pleural metastasis, primary pulmonary adenoid cystic carcinoma, solid-variant, SOX10

An incorrect **Funding** statement was provided. The correct **Funding** statement reads:

“The author(s) declared that financial support was received for this work and/or its publication. This work was supported by the Shaanxi Provincial Key Research and Development Program (General Project in the Social Development Field; Grant No. 2022SF-094) and the Xi’an Science and Technology Plan Project (Xi’an Innovation Capacity Strengthening Program, Medical Research Project, General Project; Grant No. 2021JH-03-0290).”

The original version of this article has been updated.

