# Supplementary material for: Solid-variant primary pulmonary adenoid cystic carcinoma with pleural metastasis and malignant pleural effusion: a rare case report
Source: Front Oncol. 2026 May 18;16:1827108. doi: 10.3389/fonc.2026.1827108 (PMC13222787; doi:10.3389/fonc.2026.1827108)
Supplement: Supplementary file 1 [file DataSheet1.pdf]

Supplementary Table S1. Available immunohistochemical staining information

Antibody	Clone	Dilution / Concentration	Retrieval Method	Positive Control Tissue
In-house initial diagnostic panel				
CD56	123C3	Ready-to-use	HIER, EDTA buffer, pH 9.0	Small cell lung carcinoma
Chromogranin A (CgA)	DAK-A3	Ready-to-use	HIER, EDTA buffer, pH 9.0	Appendix
Cytokeratin 7 (CK7)	OV-TL 12/30	Ready-to-use	HIER, EDTA buffer, pH 9.0	Lung adenocarcinoma
Cytokeratin 5/6 (CK5/6)	D5/16 B4	Ready-to-use	HIER, EDTA buffer, pH 9.0	Tonsil
Ki-67	MIB-1	Ready-to-use	HIER, EDTA buffer, pH 9.0	Tonsil
Napsin A	IP64	Ready-to-use	HIER, EDTA buffer, pH 9.0	Kidney
p40	BC28	Ready-to-use	HIER, EDTA buffer, pH 9.0	Lung squamous cell carcinoma
p63	DAK-p63	Ready-to-use	HIER, EDTA buffer, pH 9.0	Tonsil
Synaptophysin (Syn)	DAK-SYNAP	Ready-to-use	HIER, EDTA buffer, pH 9.0	Colon
Thyroid Transcription Factor-1 (TTF-1)	8G7G3/1	Ready-to-use	HIER, EDTA buffer, pH 9.0	Thyroid
Outside-institution extended / additional markers				
SOX10	EP268	1:100	HIER, EDTA buffer, pH 9.0	Salivary gland
c-Myb	EP769Y	1:200	HIER, EDTA buffer, pH 9.0	Tonsil
CD117 (c-KIT)	YR145	1:100	HIER, EDTA buffer, pH 9.0	Gastrointestinal stromal tumor (GIST)
Smooth Muscle Actin (SMA)	1A4	1:100	Protease retrieval	Blood vessels
Calponin	CALP	1:100	HIER, EDTA buffer, pH 9.0	Breast (myoepithelial cells)
S100 Protein	Polyclonal	1:1000	HIER, EDTA buffer, pH 9.0	Nerve
Glial Fibrillary Acidic Protein (GFAP)	Polyclonal	1:500	HIER, EDTA buffer, pH 9.0	Brain tissue
p53	DO-7	Ready-to-use	HIER, EDTA buffer, pH 9.0	Known p53-positive tumor tissue
Cytokeratin 8/18 (CK8/18)	5D3	1:100	HIER, EDTA buffer, pH 9.0	Liver

Abbreviations: HIER, heat-induced epitope retrieval; GIST, gastrointestinal stromal tumor.

Note. All in-house immunohistochemical stains were performed using the EnVision two-step detection system. The extended and additional markers were performed at an outside institution using a two-step immunohistochemical method. Original image magnification for the outside-institution immunohistochemical images shown in the manuscript (SOX10, c-Myb, CD117, S100, and SMA) was ×200.
